# Impact of renal function on residual platelet reactivity and clinical outcomes in patients with acute coronary syndrome treated with clopidogrel

**DOI:** 10.1002/clc.23588

**Published:** 2021-05-12

**Authors:** Qing Li, Yinong Chen, Ying Liu, Luyao Yu, Jingang Zheng, Yihong Sun

**Affiliations:** ^1^ Department of Cardiology Peking University China‐Japan Friendship School of Clinical Medicine Beijing 100029 China; ^2^ Department of Cardiology China–Japan Friendship Hospital Beijing 100029 China

**Keywords:** acute coronary syndrome, chronic kidney disease, high residual platelet reactivity, thromboelastography

## Abstract

**Background:**

Chronic kidney disease (CKD) is a common comorbidity in patients with acute coronary syndrome (ACS) and may potentially influence platelet function.

**Hypothesis:**

We explored the influence of renal function on platelet reactivity to investigate whether high residual platelet reactivity (HRPR) is associated with cardiovascular events.

**Methods:**

ACS patients treated with aspirin and clopidogrel were prospectively enrolled. Patients were categorized into two groups on the basis of baseline estimated glomerular filtration rate (eGFR): non‐CKD (eGFR ≥60 mL/min/1.73 m^2^) and CKD (eGFR <60 mL/min/1.73 m^2^). Platelet function was measured by thromboelastography ≥5 days after maintenance dual antiplatelet therapy. Major adverse clinical events (MACEs) were collected at 1 year after discharge.

**Results:**

There were 282 non‐CKD patients and 212 CKD patients. A significant difference in median MA_ADP_ value was observed between the two groups (15.0 mm vs. 31.3 mm, p < .001). HRPR was more prevalent in the CKD group than the non‐CKD group (27.4% vs 9.6%, p < .001). At 1‐year follow‐up, the incidence of MACEs was significantly higher for those with both CKD and HRPR compared with those with either CKD or HRPR (37.9% vs. 18.5%, p < .001). The relationship between HRPR and MACEs was consistent across CKD strata without evidence of interaction. Adding platelet reactivity to eGFR improved the model with area under the curve increasing from 0.703 to 0.734.

**Conclusion:**

In patients with ACS, the risk of HRPR increased with declining eGFR. Both CKD and HRPR were associated with MACEs at 1‐year follow‐up.

## 1 INTRODUCTION

Patients with coronary artery disease (CAD) and impaired renal function have a poor prognosis.^1^, ^2^ Despite the presence of strong epidemiological links between renal impairment and risk of thrombosis, underlying causal, and mechanistic insights have not been fully elucidated. Dual antiplatelet therapy (DAPT) including a P2Y_12_ inhibitor and aspirin is standard treatment after acute coronary syndrome (ACS) or coronary drug‐eluting stent implantation.^3^, ^4^ More potent P2Y_12_ inhibitors have been shown to be more effective for reducing the risk of ischemic events, but are associated with a higher risk of bleeding events compared with clopidogrel. Clopidogrel thus remains a treatment option for preventing ischemia events and is widely used in patients with ACS, especially those with high‐risk bleeding.^5^, ^6^ However, inadequate platelet inhibition is associated with a higher rate of ischemic events because of variations in response to clopidogrel,^7^, ^8^ with poorer response associated with CYP2C19 variant, gender, age, presence of diabetes mellitus, drug–drug interactions, and impaired renal function.^9^, ^10^, ^11^ In ACS patients with low responsiveness to clopidogrel, one study demonstrated that thromboelastography (TEG)‐guided antiplatelet therapy was shown to reduce the rate of a composite endpoint of all‐cause death, target vessel revascularization, stroke, and myocardial infarction (MI).^12^ Therefore, identifying predictors of impaired response to clopidogrel is beneficial for patients at high risk of thrombosis, especially for East Asian patients who may have a higher prevalence of CYP2C19 loss‐of‐function variants and a worse prognosis when treated with clopidogrel.^13^

The correlation between high residual platelet reactivity (HRPR) and renal function remains controversial. To date, studies evaluating the relationship between chronic kidney disease (CKD) and HRPR have been restricted by modest sample size, limited follow‐up, and inadequate multivariable adjustment.^14^, ^15^, ^16^ Moreover, for patients with CKD, it remains unclear whether low response to clopidogrel has an implication on cardiovascular events.^15^, ^17^, ^18^ We therefore investigated the influence of renal dysfunction on residual platelet reactivity and evaluated whether HRPR is related to higher incidence of adverse cardiovascular events in ACS patients with CKD.

## 2 METHODS

### 2.1 Study population

This single‐center prospective cohort study was carried out at the China–Japan Friendship Hospital from January 2015 to March 2019. A total of 2341 patients with ACS who were treated with DAPT and underwent TEG were screened for eligibility. The inclusion criteria were age ≥ 18 years, hospitalization for ACS with or without percutaneous coronary intervention (PCI), and treatment with DAPT included aspirin and clopidogrel. Exclusion criteria were duration of clopidogrel and aspirin treatment <5 days, treatment with oral anticoagulants, concomitant administration of glycoprotein IIb/IIIa inhibitors, and treatment with non‐steroidal anti‐inflammatory drugs or glucocorticoids. The study protocol was approved by the Ethics Committee of the China–Japan Friendship Hospital. This cohort study was conducted in accordance with the principles of the Declaration of Helsinki. Written informed consent was obtained from all patients prior to participation.

### 2.2 Data collection

Demographic parameters and comorbidities were collected from patients' medical records, including gender, age, current smoking status, body mass index, and history of hyperlipidemia, hypertension, diabetes mellitus, stroke, myocardial infarction, and prior revascularization. Clinical characteristics including left ventricular ejection fraction, laboratory test results, and medications at discharge were also collected. Baseline serum creatinine levels were measured on the first day of admission. The estimated glomerular filtration rate (eGFR) was calculated using the Modification of Diet in Renal Disease (MDRD) formula^19^: eGFR (mL/min/1.73 m^2^) = 175 × S_Cr_^‐1.154^ × age^‐0.203^ × 0.742 (if female) × 1.212 (if black). Patients with eGFR <60 mL/min/1.73 m^2^ were diagnosed as having CKD in accordance with the Kidney Disease: Improving Global Outcomes (KDIGO) guideline.^20^

### 2.3 Platelet function assessment

Platelet reactivity was assessed using TEG (LEPU Medical Technology Co., Ltd, Beijing, China), a convenient method to evaluate the contributions of platelet and fibrin to clot strength. Maximum amplitude (MA), as an indicator of the strength of the final clot, is the parameter directly measured using this method. MA_ADP_ (adenosine diphosphate) represents residual platelet reactivity in the ADP pathway. For MA_ADP_ measurement, blood samples were collected between 6 and 12 hours after routine administration of antiplatelet medication. Platelet function was evaluated using TEG within 2 hours of blood drawing, in accordance with the manufacturer's instructions. A 360‐μL blood sample was added to a heparinase‐coated cup containing 100 μL ADP (2 μmol/L) to generate a whole blood‐crosslinked clot with platelet activation. On the basis of prior studies, we defined HRPR as MA_ADP_ > 47 mm in the present study.^21^, ^22^

### 2.4 Clinical outcomes

Major adverse clinical events (MACEs) and bleeding events at 1‐year follow‐up were defined as the clinical outcomes. MACEs were a composite endpoint of all‐cause death, ischemic stroke, and MI. MI was defined as type I MI in accordance with the fourth universal definition of MI,^23^ and represented the ischemic symptoms with abnormal electrocardiographic and elevated troponin. Computed tomography or magnetic resonance imaging of the head were adopted to diagnose ischemic stroke. Major bleeding event was defined as Bleeding Academic Research Consortium Definition (BARC) type 3 or 5.^24^ All patients were followed up by telephone after discharge.

### 2.5 Statistical analysis

Continuous variables were expressed as means ± standard deviations or medians with interquartile ranges. Student's *t* test or the Mann–Whitney U test were used to compare continuous variables between groups. Categorical variables were shown as numbers (percentages) and compared using the Chi‐squared test or Fisher's exact test. For continuous variables, the Kolmogorov–Smirnov test was used to examine normal distribution. The odds ratio (OR) or hazard ratio (HR) was displayed with 95% confidence intervals (CIs). All patients were categorized into either a non‐CKD group (eGFR ≥60 mL/min/1.73 m^2^) or a CKD group (eGFR <60 mL/min/1.73 m^2^) as there was no significant difference in MA_ADP_ observed between those with severe renal dysfunction (eGFR <30 mL/min/1.73 m^2^) and moderate renal dysfunction (30 mL/min/1.73 m^2^ ≤ eGFR <60 mL/min/1.73 m^2^). Independent risk factors for HRPR on clopidogrel were examined using a multivariate logistic regression analysis model. If values of p < .05 were observed between the group with HRPR and the group without HRPR in the univariate regression analysis model, variables were selected. In the absence of differences between the group with HRPR and the group without HRPR, previously reported factors related to influence platelet function including diabetes mellitus, clinical presentation, proton‐pump inhibitors (PPIs), and PCI were entered into the model.^10^, ^25^, ^26^, ^27^, ^28^ The following confounders were adjusted: age > 65 years, female gender, hypertension, diabetes mellitus, hemoglobin <100 g/L, clinical presentation, PCI, PPIs, and CKD. Kaplan–Meier curves were used to calculate time to ischemic and bleeding events among patients (a) with and without CKD or (b) with or without CKD stratified by HRPR on clopidogrel. Survival curves were compared using the log‐rank test across the CKD group and the non‐CKD group. We evaluated whether an interaction was present for the influence of CKD and HRPR on clinical outcomes. A Cox proportional hazards regression model was used to identify prognostic factors. Variables with values of p < .05 in the univariate analysis were selected for multivariate analysis, as were those previously reported to have an impact on MACEs.^28^ Receiver operating characteristic (ROC) curve analyses were performed to quantify the ability of eGFR and MA_ADP_ to predict MACEs using the R version 4.0.3 (R Foundation for Statistical Computing, Vienna, Austria). All statistical analyses were performed using the SPSS version 25.0 (SPSS Inc., Chicago, IL). Graphical presentations were generated using the Prism version 8.2.1 (GraphPad Software Inc., La Jolla, California). A two‐tailed value of p < .05 was considered statistically significant.

## 3 RESULTS

### 3.1 Baseline characteristics

**FIGURE 1:**
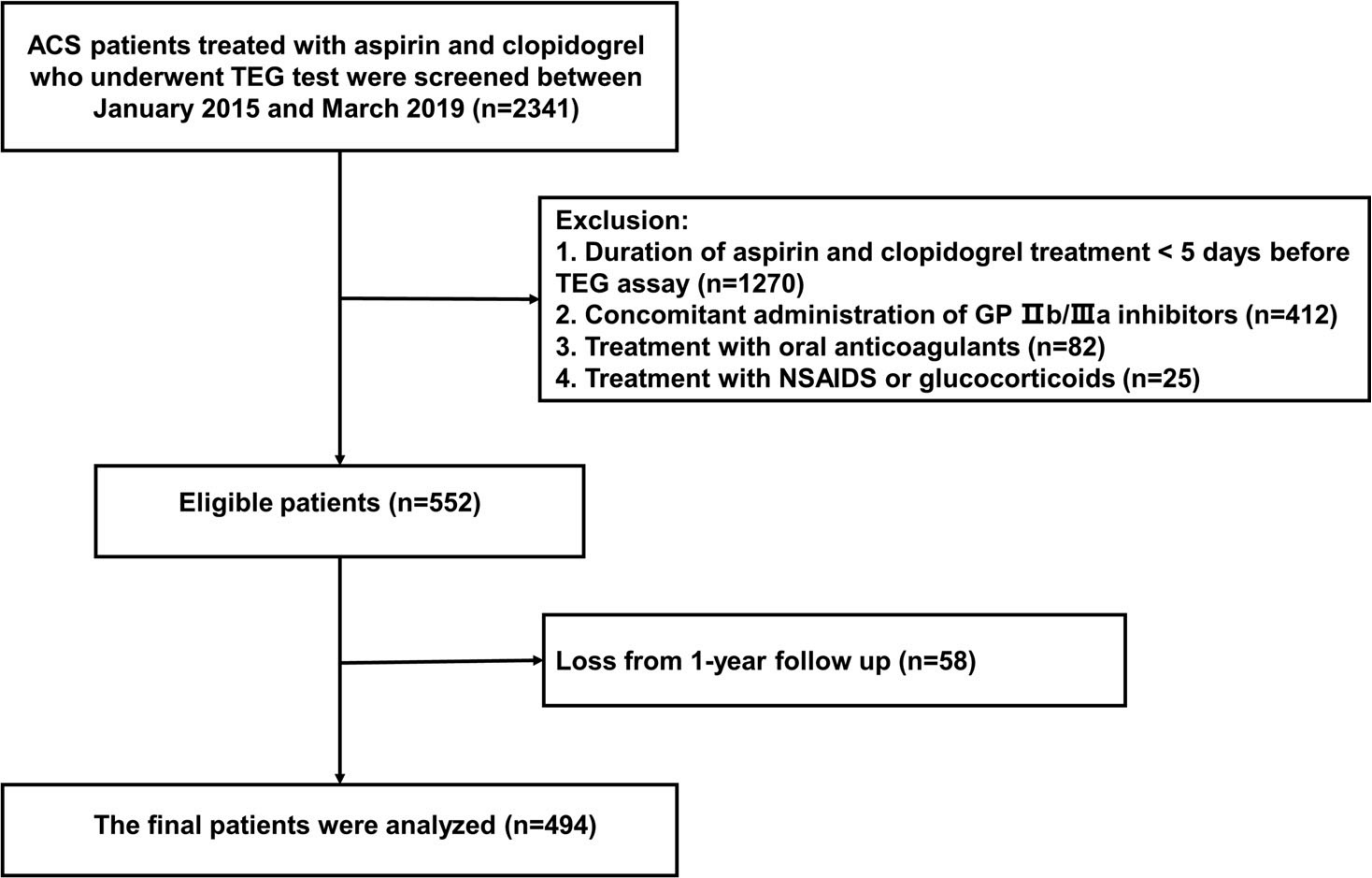
Patient flow chart for the study cohort. ACS, acute coronary syndrome; GP IIb/IIIa inhibitors, glycoprotein IIb/IIIa inhibitors; NSAIDS, non‐steroidal anti‐inflammatory drugs; TEG, thromboelastography

A total of 2341 ACS patients treated with DAPT who underwent TEG were screened, of which 494 patients were eligible for inclusion in the present analysis (Figure 1). The average duration from the initiation of DAPT treatment to TEG was 6 ± 1 days during hospitalization. There were 282 patients (57.1%) in the non‐CKD group (eGFR ≥60 mL/min/1.73 m^2^) and 212 patients (42.9%) in the CKD group (eGFR <60 mL/min/1.73 m^2^). In addition, the CKD group included 95 patients with severe renal dysfunction (eGFR <30 mL/min/1.73 m^2^), of whom 52 patients were receiving dialysis. The baseline characteristics of included patients are shown in Table 1. Patients in the CKD group were significantly older and more likely to have comorbidities, including hypertension, diabetes mellitus, history of stroke, and history of coronary artery bypass grafting but were less likely to be smokers compared with patients in the non‐CKD group. CKD patients were also less likely to receive angiotensin‐converting enzyme inhibitors or angiotensin receptor blockers but were more likely to use calcium‐channel blockers and PPIs. There were more patients with non‐ST‐segment elevation myocardial infarction in the CKD group compared with the non‐CKD group. Furthermore, patients in the CKD group were less likely to receive PCI.

**TABLE 1:** Baseline demographics and clinical characteristics

Variables	Non‐CKD (n = 282)	CKD (n = 212)	*p* value
Clinical characteristics			
Age (years)	68 (60–78)	74 (64–81)	<.001
Female gender	105 (37.2%)	87 (41.0%)	.391
BMI (kg/m^2^)	25.3 ± 3.7	24.4 ± 4.0	.004
Current smoking	91 (32.3%)	44 (20.8%)	.004
Hypertension	201 (71.3%)	186 (87.7%)	<.001
Diabetes mellitus	130 (46.1%)	122 (57.5%)	.012
Hyperlipidemia	181 (64.2%)	145 (68.4%)	.328
Prior stroke	53 (18.8%)	67 (31.6%)	.001
Prior MI	42 (14.9%)	44 (20.8%)	.089
Prior PCI	69 (24.5%)	48 (22.6%)	.636
Prior CABG	6 (2.1%)	18 (8.5%)	.001
Prior bleeding	24 (8.5%)	27 (12.7%)	.127
Laboratory test			
Hemoglobin (g/L)	132 (122–146)	115 (98–129)	<.001
Platelet (10^9^/L)	200 (165–236)	189 (154–229)	.017
Mean platelet volume (fL)	10.0 ± 1.4	10.4 ± 1.4	<.001
LDL‐C (mmol/L)	2.5 ± 0.9	2.6 ± 1.0	.488
eGFR (mL/min/1.73 m2)	90.3 (76.1–107.2)	35.7 (11.6–48.3)	<.001
LVEF (%)	61 (52–69)	55 (45–65)	<.001
LVEF<50 (%)	52 (18.4%)	77 (36.3%)	<.001
Clinical presentation			.006
Unstable angina	136 (48.2%)	73 (34.4%)	
NSTEMI	72 (25.5%)	76 (35.8%)	
STEMI	74 (26.2%)	63 (29.7%)	
PCI	213 (75.5%)	131 (61.8%)	.001
Medication at discharge			
ACEI or ARB	173 (61.3%)	77 (36.3%)	<.001
β‐blocker	230 (81.6%)	186 (87.7%)	.062
Statins	279 (98.9%)	209 (98.6%)	1.000
CCB	35 (12.4%)	88 (41.5%)	<.001
PPIs	159 (56.4%)	159 (75.0%)	<.001

*Note*: Continuous data are presented as means ± standard deviation (SD) or median (interquartile range) and categorical data was shown as n (%). p value in this table was analyzed between 2 groups.

Abbreviations: ACEI, angiotensin‐converting enzyme inhibitor; ARB, angiotensin receptor blocker; BMI, body mass index; CABG, coronary artery bypass grafting; CCB, calcium‐channel blocker; CKD, chronic kidney disease; eGFR, estimate glomerular filtration; MI, myocardial infarction; LDL‐C, low‐density lipoprotein cholesterol; LVEF, left ventricular ejection fraction. NSTEMI, non‐ST‐segment elevation myocardial infarction; PCI, percutaneous coronary intervention; PPIs, proton pump inhibitors; STEMI, ST‐segment elevation myocardial infarction.

### 3.2 Renal function and platelet reactivity

A significant but weak correlation was observed between MA_ADP_ and eGFR (r = −0.344, p < .001). The median MA_ADP_ value was higher in the CKD group compared with the non‐CKD group (31.3 mm vs. 15.0 mm, p < .001). Compared with patients with normal renal function, the incidence of HRPR occurred more frequently in those with renal dysfunction (27.4% vs. 9.6%, p < .001). The univariate logistic regression analysis model showed that there were more HRPR patients with age > 65 years, female gender, hypertension, hemoglobin <100 g/L, and CKD compared with patients without HRPR (Table S1). Multivariate logistic regression analysis showed that CKD and female gender were significantly related to HRPR in response to clopidogrel (Table 2).

**TABLE 2:** Risk factors for HRPR on clopidogrel by univariate and multivariate logistic regression analysis model

Variables	Univariate		Multivariate	
	OR (95% CI)	*p* value	OR (95% CI)	*p* value
Age > 65 (years)	2.28 (1.32–3.94)	.003	1.72 (0.95–3.13)	.074
Female gender	3.03 (1.87–4.90)	<.001	2.83 (1.69–4.75)	<.001
Hypertension	2.06 (1.05–4.05)	.035	1.47 (0.72–3.00)	.290
Diabetes mellitus	1.55 (0.97–2.50)	.070	1.24 (0.74–2.09)	.421
Hemoglobin <100 (g/L)	2.76 (1.52–5.01)	.001	1.29 (0.65–2.56)	.468
Presentation				
Unstable angina	Reference		Reference	
NSTEMI	0.96 (0.55–1.70)	.894	0.73 (0.39–1.35)	.310
STEMI	1.16 (0.67–2.04)	.594	1.10 (0.58–2.10)	.766
PCI	0.76 (0.47–1.25)	.278	1.10 (0.63–1.93)	.745
PPIs	1.50 (0.90–2.51)	.119	0.94 (0.53–1.66)	.821
CKD	3.56 (2.16–5.86)	<.001	3.17 (1.78–5.63)	<.001

*Note*: Multivariate logistic regression analysis model included age > 65 years, female gender, hypertension, diabetes mellitus, hemoglobin <100 (g/L), clinical presentation, PCI, PPIs, and CKD.

Abbreviations: CI, confidence interval; HRPR, high residual platelet reactivity; NSTEMI, acute non‐ST‐segment elevation myocardial infarction; PCI, percutaneous coronary intervention; PPIs, proton pump inhibitors; OR, odds ratio; STEMI, acute ST‐segment elevation myocardial infarction.

### 3.3 MACEs and bleeding events at 1 year

**FIGURE 2:**
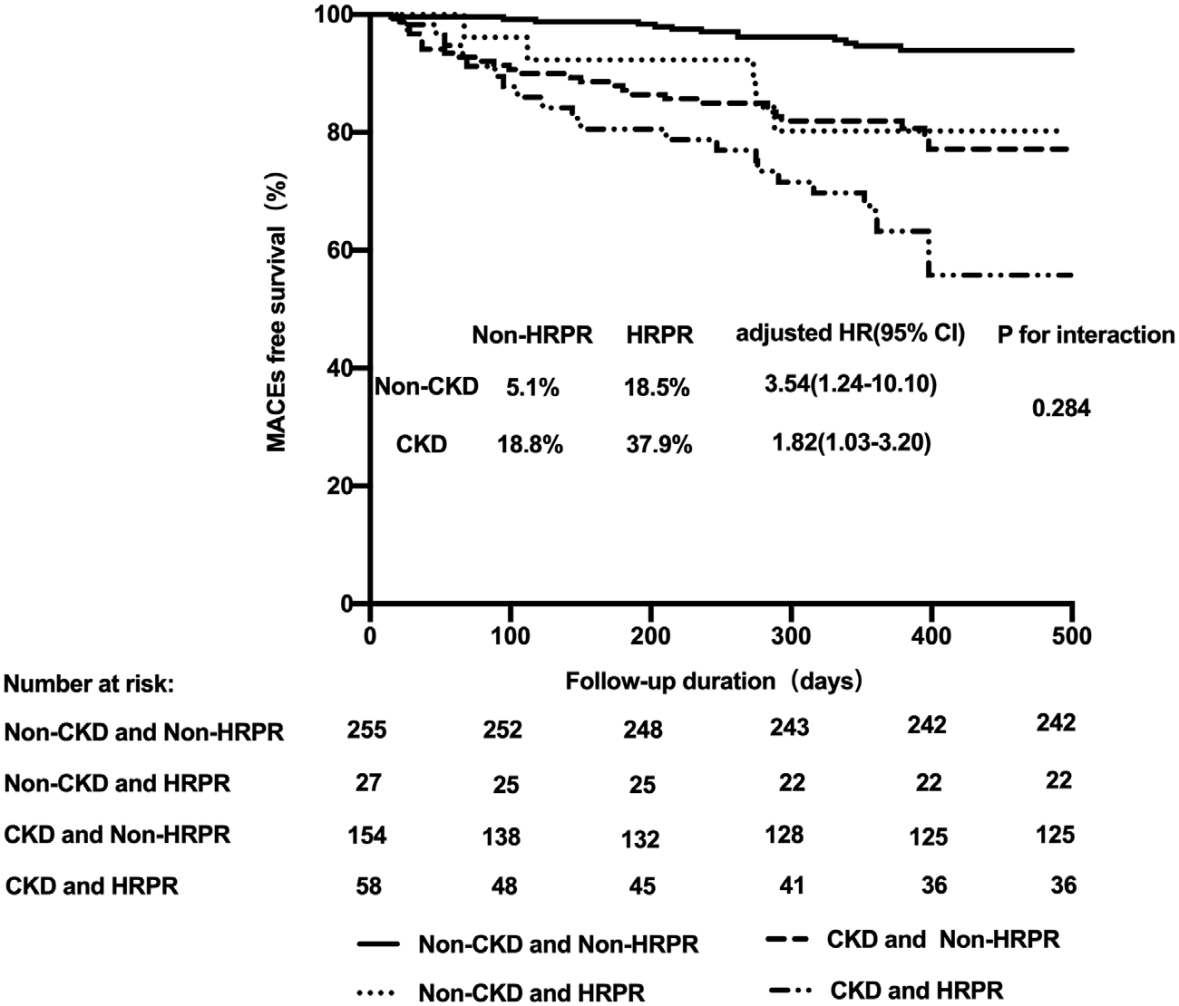
Kaplan–Meier curves for MACEs based on non‐HRPR versus HRPR status in patients with or without CKD. MACEs, major adverse clinical events; HRPR, high residual platelet reactivity; CKD, chronic kidney disease

**FIGURE 3:**
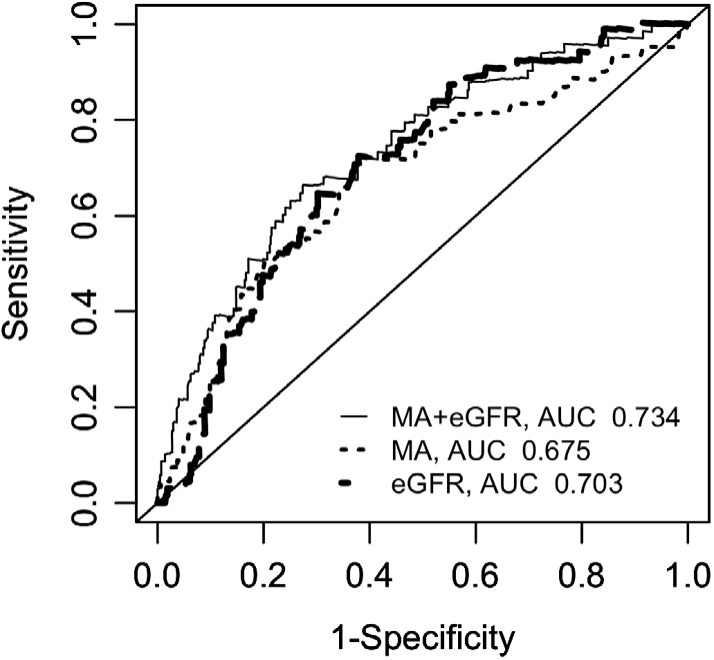
ROC for predicting the risk of MACEs. eGFR, estimated glomerular filtration rate; MA, maximum amplitude; MACEs, major adverse clinical events; ROC, receiver operating characteristic curve

A total of 69 MACEs were observed (31 deaths, 11 ischemic stroke events, and 27 MI events) over 1 year of follow‐up. The incidence of MACEs was significantly higher in the CKD group than in the non‐CKD group at follow‐up (24.1% vs. 6.4%, log‐rank p < .001). Patients with renal dysfunction also experienced more bleeding events compared with those without CKD (6.6% vs. 2.1%, log‐rank p = .007). The incidence of MACEs was significantly higher in HRPR patients compared with those without HRPR, irrespective of CKD status (Table S2). However, no relationship was observed between the presence of HRPR and bleeding events in patients with or without CKD. The univariate Cox regression analysis model showed that age (per 10 years), hypertension, prior stroke, Prior MI, HRPR, and CKD were associated with MACEs (Table S3). Multivariate Cox regression analysis identified that CKD (HR = 2.88, 95%CI 1.63–5.10, p < .001) and HRPR (HR = 2.06, 95%CI 1.24–3.41, p = .005) were independently associated with 1‐year MACEs (Table S4). Furthermore, older age and prior MI were significantly associated with the risk of MACEs. There was no significant interaction between HRPR and CKD for the risk of MACEs (p for interaction = .284)

(Figure 2). Regarding the prediction of any MACEs, area under the curve for eGFR was 0.703 with sensitivity and specificity of 0.739 and 0.624, and was 0.675 for platelet reactivity with sensitivity and specificity of 0.710 and 0.616. Adding platelet reactivity to eGFR improved the model, with area under the curve increasing from 0.703 to 0.734 (Figure 3).

## 4 DISCUSSION

The findings of this study provide important information about the impact of renal dysfunction on residual platelet reactivity and clinical outcomes in ACS patients receiving clopidogrel. Firstly, we showed that the prevalence of HRPR was more common in CKD patients in this cohort study, including patients on dialysis. Secondly, renal dysfunction was associated with a higher risk of mortality, ischemic events, and bleeding events among ACS patients with or without PCI. Thirdly, HRPR was shown to be an independent risk factor for MACEs, regardless of renal function, with the highest risk observed among patients with both CKD and HRPR at 1‐year follow‐up.

Renal dysfunction was associated with a higher incidence of HRPR, which aligned with previous studies using a variety of platelet function tests.^15^, ^16^, ^25^ Guo et al. showed that renal impairment was associated with higher residual platelet reactivity measured using the VerifyNow P2Y_12_ assay in Korean PCI patients receiving DAPT.^16^ Angiolillo et al. also demonstrated that impaired renal function was related to HRPR using light transmittance aggregometry in patients with diabetes on maintenance DAPT for at least 30 days.^25^ However, in a study of stable CAD patients, no impact of renal dysfunction was observed on HRPR measured using the vasodilator‐stimulated phosphoprotein phosphorylation test.^29^ However, there are some important considerations when interpreting these findings, such as a lack of uniformity in the time point of platelet reactivity measurement. Most studies on platelet function use one assay and single time point measurement early after initiation of clopidogrel therapy. Platelet reactivity varies depending on duration of clopidogrel treatment, and tends to stabilize after several weeks of treatment. For example, in the Assessment of Dual Antiplatelet Therapy with Drug‐Eluting Stents Registry study (ADAPT‐DES), the higher prevalence of HRPR was partly explained by the average test time point of 20 hours after PCI^17^. Wide variability is observed in early pharmacodynamic responses after loading doses of clopidogrel, especially in patients with ACS. Moreover, the proportion of patients with severe renal dysfunction was relatively low in previous studies,^17^, ^30^ leading to underestimation of the prognostic utility of platelet function test in this high‐risk population. Rubin et. al showed that nearly two‐thirds of CKD patients on hemodialysis exhibited HRPR with clopidogrel.^31^ Furthermore, the proportion of inadequate platelet inhibition in CKD stage 5 patients has been shown to be higher than in those with moderate renal dysfunction.^32^ Our study enrolled a considerable proportion of patients with end‐stage CKD, including 52 patients (10.5%) on dialysis.

The relationship between HRPR and clinical outcomes in CKD patients remains controversial. The findings from two previous studies of patients with stable CAD or PCI showed that there was no significant association between HRPR and MACEs among PCI patients with and without CKD.^15^, ^29^ Morel et al. reported that HRPR was associated with a higher rate of MACEs in CKD patients, but not in those without CKD in a cohort study of patients receiving urgent or planned PCI^18^. A sub‐study of ADAPT‐DES showed that HPRR and adverse clinical events remained significantly correlated irrespective of CKD status^17^. The mechanisms for increased risk of MACEs in CKD patients are complicated. High levels of tissue factor and fibrinogen may lead to activation of the coagulation cascade, increasing blood thrombogenicity in the uremic milieu.^33^, ^34^ In addition, atherosclerotic plaques have been found to be consisted of a greater proportion of necrotic core and less fibrous tissue in CKD patients, which may increase plaque vulnerability.^35^ Furthermore, CKD patients have been shown to exhibit increased platelet activity.^14^, ^25^

This disconcerting finding raises the question of whether antiplatelet dose regimen adjustments should be considered in HRPR patients, a strategy not recommended in current guidelines. In addition, CKD patients are typically underrepresented or excluded from clinical trials. A post‐hoc analysis of the CREDO trial indicated that patients with mild or moderate renal dysfunction treated with clopidogrel did not obtain the same benefit as patients with preserved kidney function.^36^ Whether patients with impaired renal function should be treated with more potent antiplatelet agents remains to be established. Compared with CKD patients receiving clopidogrel, a previous observational study reported a trend toward lower incidence of MI reoccurrence in those treated with prasugrel at 1‐year follow‐up^37^. A sub‐analysis of the PLATO trial showed that ACS patients with CKD treated with ticagrelor had a greater reduction in ischemic events compared with non‐CKD patients, without a significant increase in major bleeding events.^38^ A single‐center study showed that ticagrelor resulted in faster and greater platelet inhibition compared with clopidogrel in patients with kidney failure receiving hemodialysis.^39^ Although low‐dose prasugrel had a better antiplatelet effect than clopidogrel, it did not significantly improve the prevalence of HRPR in a study of Japanese hemodialysis patients.^40^ However, randomized clinical trials of the use of platelet function tests to tailor antiplatelet therapy have failed to demonstrate any benefit, and were largely limited by the inclusion of low‐risk populations. Baber et al. suggested that additional risk markers may potentially allow clinicians to stratify patients to optimal antithrombotic therapy in clinical practice.^17^ We suggest that further studies are needed to assess the benefit and risk of administering more potent P2Y_12_ receptor antagonists to ACS patients with CKD.

## 5 LIMITATIONS

The present study had several limitations, including the small sample size and single‐center observational study design, which may have contributed to selection bias. Our study also had a relatively high proportion of patients with severe renal dysfunction who exhibited higher platelet reactivity than patients without CKD.^32^, ^41^ Additionally, platelet reactivity was assessed only at a single time point, despite all patients having already taken clopidogrel for at least 5 days. Meanwhile, serum creatinine was evaluated only at admission or before PCI, although CKD status can change, especially in those receiving PCI for contrast‐related renal dysfunction. Furthermore, we did not evaluate the impact of the CYP2C19 genotype on HRPR, although prior studies had reported that CYP2C19 genotype can partly explain the variability observed in response to clopidogrel.^42^, ^43^ Moreover, we were unable to confirm whether compliance with DAPT was adequate during follow‐up as the clopidogrel active metabolite was not measured regularly. Finally, we did not collect data on new P2Y12 inhibitors such as prasugrel or ticagrelor, given their limited use in patients requiring antiplatelet therapy, especially those with CKD.

## 6 CONCLUSION

Our findings demonstrated that renal dysfunction was associated with residual platelet reactivity and a high risk of 1‐year MACEs in ACS patients treated with clopidogrel. HRPR was associated with risk of MACEs at 1‐year follow up, independent of renal function. Antiplatelet treatment strategies in ACS patients with severe CKD require optimization using further studies.

## CONFLICT OF INTEREST

The authors declare no potential conflict of interest.

## Supporting information


**Table S1** The incidence of clinical outcomes in patients with or without CKD, stratified by the HRPR on clopidogrel.
**Table S2:** Risk factors for MACEs by multivariate Cox regression analysis model.
**Table S3:** Risk factors for HRPR on clopidogrel by univariate logistic regression analysis model
**Table S4:** Risk factors for MACEs by univariate Cox regression analysis model.

## Data Availability

The dataset analyzed during the current study is available from the corresponding author on reasonable request.

## References

[1] US renal data system 2019 annual data report: epidemiology of kidney disease in the United States Am J Kidney Dis, 75, 2020;(1 Suppl 1):A6‐A7. 10.1053/j.ajkd.2019.09.003 31704083

[2] Tonelli M , Karumanchi SA , Thadhani R . Epidemiology and mechanisms of uremia‐related cardiovascular disease. Circulation. 2016;133(5):518‐536. 26831434 10.1161/CIRCULATIONAHA.115.018713

[3] Bonello L , Angiolillo DJ , Aradi D , Sibbing D . P2Y‐ADP receptor blockade in chronic kidney disease patients with acute coronary syndromes. Circulation. 2018;138(15):1582‐1596. 30354508 10.1161/CIRCULATIONAHA.118.032078

[4] Neumann FJ , Sousa‐Uva M , Ahlsson A , et al. 2018 ESC/EACTS guidelines on myocardial revascularization. Eur Heart J. 2019;40(2):87‐165. 30615155 10.1093/eurheartj/ehy855

[5] Nanna MG , Granger CB . In ACS, ticagrelor and prasugrel each reduce some ischemic events but increase major bleeding vs. clopidogrel. Ann Intern Med. 2020;173(8):Jc44. 33075264 10.7326/ACPJ202010200-044

[6] Szummer K , Montez‐Rath ME , Alfredsson J , et al. Comparison between ticagrelor and clopidogrel in elderly patients with an acute coronary syndrome: insights from the SWEDEHEART registry. Circulation. 2020;142(18):1700‐1708. 32867508 10.1161/CIRCULATIONAHA.120.050645

[7] Giustino G , Redfors B , Kirtane AJ , et al. Platelet reactivity and risk of ischemic stroke after coronary drug‐eluting stent implantation: from the ADAPT‐DES study. JACC Cardiovasc Interv. 2018;11(13):1277‐1286. 29908967 10.1016/j.jcin.2018.01.263

[8] Aradi D , Kirtane A , Bonello L , et al. Bleeding and stent thrombosis on P2Y12‐inhibitors: collaborative analysis on the role of platelet reactivity for risk stratification after percutaneous coronary intervention. Eur Heart J. 2015;36(27):1762‐1771. 25896078 10.1093/eurheartj/ehv104

[9] Joo HJ , Ahn SG , Park JH , et al. Effects of genetic variants on platelet reactivity and one‐year clinical outcomes after percutaneous coronary intervention: a prospective multicentre registry study. Sci Rep. 2018;8(1):1229. 29352151 10.1038/s41598-017-18134-y PMC5775197

[10] Baber U , Bander J , Karajgikar R , et al. Combined and independent impact of diabetes mellitus and chronic kidney disease on residual platelet reactivity. Thromb Haemost. 2013;110(1):118‐123. 23677380 10.1160/TH13-01-0004

[11] Jiang XL , Samant S , Lesko LJ , Schmidt S . Clinical pharmacokinetics and pharmacodynamics of clopidogrel. Clin Pharmacokinet. 2015;54(2):147‐166. 25559342 10.1007/s40262-014-0230-6 PMC5677184

[12] Tang Y‐D , Wang W , Yang M , et al. Randomized comparisons of double‐dose clopidogrel or adjunctive cilostazol versus standard dual antiplatelet in patients with high posttreatment platelet reactivity: results of the CREATIVE trial. Circulation. 2018;137(21):2231‐2245. 29420189 10.1161/CIRCULATIONAHA.117.030190

[13] Wang Y , Zhao X , Lin J , et al. Association between CYP2C19 loss‐of‐function allele status and efficacy of clopidogrel for risk reduction among patients with minor stroke or transient ischemic attack. JAMA. 2016;316(1):70‐78. 27348249 10.1001/jama.2016.8662

[14] Gremmel T , Müller M , Steiner S , et al. Chronic kidney disease is associated with increased platelet activation and poor response to antiplatelet therapy. Nephrol Dial Transplant. 2013;28(8):2116‐2122. 23729489 10.1093/ndt/gft103

[15] Zhu P , Tang X‐F , Xu J‐J , et al. Platelet reactivity in patients with chronic kidney disease undergoing percutaneous coronary intervention. Platelets. 2019;30(7):901‐907. 30518271 10.1080/09537104.2018.1549319

[16] Guo LZ , Kim MH , Shim CH , Choi SY , Serebruany VL . Impact of renal impairment on platelet reactivity and clinical outcomes during chronic dual antiplatelet therapy following coronary stenting. Eur Heart J Cardiovasc Pharmacother. 2016;2(3):145‐151. 27533756 10.1093/ehjcvp/pvv052

[17] Baber U , Mehran R , Kirtane AJ , et al. Prevalence and impact of high platelet reactivity in chronic kidney disease: results from the assessment of dual antiplatelet therapy with drug‐eluting stents registry. Circ Cardiovasc Interv. 2015;8(6):e001683. 26056248 10.1161/CIRCINTERVENTIONS.115.001683

[18] Morel O , El Ghannudi S , Jesel L , et al. Cardiovascular mortality in chronic kidney disease patients undergoing percutaneous coronary intervention is mainly related to impaired P2Y12 inhibition by clopidogrel. J Am Coll Cardiol. 2011;57(4):399‐408. 21251579 10.1016/j.jacc.2010.09.032

[19] Levey AS , Coresh J , Greene T , et al. Using standardized serum creatinine values in the modification of diet in renal disease study equation for estimating glomerular filtration rate. Ann Intern Med. 2006;145(4):247‐254. 16908915 10.7326/0003-4819-145-4-200608150-00004

[20] National Kidney Foundation . Clinical practice guideline for the evaluation and management of chronic kidney disease. Kidney Int. Suppl. 2013;3(1):1‐163.

[21] Jeong Y‐H , Bliden KP , Antonino MJ , Tantry US , Gurbel PA . Usefulness of thrombelastography platelet mapping assay to measure the antiplatelet effect of P2Y(12) receptor inhibitors and high on‐treatment platelet reactivity. Platelets. 2013;24(2):166‐169. 22646305 10.3109/09537104.2012.675108

[22] Wu H‐Y , Zhang C , Zhao X , Qian J‐Y , Wang Q‐B , Ge J‐B . Residual platelet reactivity is preferred over platelet inhibition rate in monitoring antiplatelet efficacy: insights using thrombelastography. Acta Pharmacol Sin. 2020;41(2):192‐197. 31515526 10.1038/s41401-019-0278-9 PMC7468573

[23] Thygesen K , Alpert JS , Jaffe AS , et al. Fourth universal definition of myocardial infarction (2018). Circulation. 2018;138(20):e618‐e651. 30571511 10.1161/CIR.0000000000000617

[24] Mehran R , Rao SV , Bhatt DL , et al. Standardized bleeding definitions for cardiovascular clinical trials: a consensus report from the bleeding academic research consortium. Circulation. 2011;123(23):2736‐2747. 21670242 10.1161/CIRCULATIONAHA.110.009449

[25] Angiolillo DJ , Bernardo E , Capodanno D , et al. Impact of chronic kidney disease on platelet function profiles in diabetes mellitus patients with coronary artery disease taking dual antiplatelet therapy. J Am Coll Cardiol. 2010;55(11):1139‐1146. 20223369 10.1016/j.jacc.2009.10.043

[26] Lee S , Hizoh I , Kovacs A , et al. Predictors of high on‐clopidogrel platelet reactivity in patients with acute coronary syndrome. Platelets. 2016;27(2):159‐167. 26247099 10.3109/09537104.2015.1054799

[27] Holm M , Dalén M , Tornvall P , van der Linden J . Point‐of‐care testing of clopidogrel‐mediated platelet inhibition and risk for cardiovascular events after coronary angiography with or without percutaneous coronary intervention. Blood Coagul Fibrinol. 2014;25(6):577‐584. 10.1097/MBC.0000000000000103 24614428

[28] Nicolau JC , Bhatt DL , Roe MT , et al. Concomitant proton‐pump inhibitor use, platelet activity, and clinical outcomes in patients with acute coronary syndromes treated with prasugrel versus clopidogrel and managed without revascularization: insights from the targeted platelet inhibition to clarify the optimal strategy to medically manage acute coronary syndromes trial. Am Heart J. 2015;170(4):683‐694. 26386792 10.1016/j.ahj.2015.05.017

[29] Mavrakanas TA , Alam A , Reny J‐L , Fontana P . Platelet reactivity in stable cardiovascular patients with chronic kidney disease. Platelets. 2018;29(5):455‐462. 28580812 10.1080/09537104.2017.1316485

[30] Mangiacapra F , Cavallari I , Barbato E , et al. Impact of chronic kidney disease on platelet reactivity and outcomes of patients receiving clopidogrel and undergoing percutaneous coronary intervention. Am J Cardiol. 2014;113(7):1124‐1129. 24507863 10.1016/j.amjcard.2013.12.018

[31] Rubin GA , Kirtane AJ , Chen S , et al. Impact of high on‐treatment platelet reactivity on outcomes following PCI in patients on hemodialysis: an ADAPT‐DES substudy. Catheter Cardiovasc Interv. 2020;96(4):793‐801. 31721430 10.1002/ccd.28577

[32] Muller C , Caillard S , Jesel L , et al. Association of estimated GFR with platelet inhibition in patients treated with clopidogrel. Am J Kidney Dis. 2012;59(6):777‐785. 22425260 10.1053/j.ajkd.2011.12.027

[33] Chitalia VC , Shivanna S , Martorell J , et al. Uremic serum and solutes increase post‐vascular interventional thrombotic risk through altered stability of smooth muscle cell tissue factor. Circulation. 2013;127(3):365‐376. 23269489 10.1161/CIRCULATIONAHA.112.118174 PMC4407990

[34] Shlipak MG , Fried LF , Crump C , et al. Elevations of inflammatory and procoagulant biomarkers in elderly persons with renal insufficiency. Circulation. 2003;107(1):87‐92. 12515748 10.1161/01.cir.0000042700.48769.59

[35] Baber U , Stone GW , Weisz G , et al. Coronary plaque composition, morphology, and outcomes in patients with and without chronic kidney disease presenting with acute coronary syndromes. JACC Cardiovasc Imaging. 2012;5(3 Suppl):S53‐S61. 22421231 10.1016/j.jcmg.2011.12.008

[36] Best PJM , Steinhubl SR , Berger PB , et al. The efficacy and safety of short‐ and long‐term dual antiplatelet therapy in patients with mild or moderate chronic kidney disease: results from the clopidogrel for the reduction of events during observation (CREDO) trial. Am Heart J. 2008;155(4):687‐693. 18371477 10.1016/j.ahj.2007.10.046

[37] Baber U , Chandrasekhar J , Sartori S , et al. Associations between chronic kidney disease and outcomes with use of Prasugrel versus clopidogrel in patients with acute coronary syndrome undergoing percutaneous coronary intervention: a report from the PROMETHEUS study. JACC Cardiovasc Interv. 2017;10(20):2017‐2025. 28780028 10.1016/j.jcin.2017.02.047

[38] James S , Budaj A , Aylward P , et al. Ticagrelor versus clopidogrel in acute coronary syndromes in relation to renal function: results from the platelet inhibition and patient outcomes (PLATO) trial. Circulation. 2010;122(11):1056‐1067. 20805430 10.1161/CIRCULATIONAHA.109.933796

[39] Jeong KH , Cho JH , Woo JS , et al. Platelet reactivity after receiving clopidogrel compared with ticagrelor in patients with kidney failure treated with hemodialysis: a randomized crossover study. Am J Kidney Dis. 2015;65(6):916‐924. 25622774 10.1053/j.ajkd.2014.11.023

[40] Ohno Y , Kitahara H , Fujii K , et al. High residual platelet reactivity after switching from clopidogrel to low‐dose prasugrel in Japanese patients with end‐stage renal disease on hemodialysis. J Cardiol. 2019;73(1):51‐57. 30055865 10.1016/j.jjcc.2018.07.001

[41] Alexopoulos D , Xanthopoulou I , Panagiotou A , et al. Prevalence of inadequate platelet inhibition by clopidogrel in patients receiving hemodialysis. Am J Kidney Dis. 2012;59(3):469‐471. 22154328 10.1053/j.ajkd.2011.10.045

[42] Hochholzer W , Trenk D , Fromm MF , et al. Impact of cytochrome P450 2C19 loss‐of‐function polymorphism and of major demographic characteristics on residual platelet function after loading and maintenance treatment with clopidogrel in patients undergoing elective coronary stent placement. J Am Coll Cardiol. 2010;55(22):2427‐2434. 20510210 10.1016/j.jacc.2010.02.031

[43] Shuldiner AR , O'Connell JR , Bliden KP , et al. Association of cytochrome P450 2C19 genotype with the antiplatelet effect and clinical efficacy of clopidogrel therapy. JAMA. 2009;302(8):849‐857. 19706858 10.1001/jama.2009.1232 PMC3641569

