# Supplementary material for: Impact of renal function on residual platelet reactivity and clinical outcomes in patients with acute coronary syndrome treated with clopidogrel
Source: Clin Cardiol. 2021 May 12;44(6):789–96. doi: 10.1002/clc.23588 (PMC8207985; doi:10.1002/clc.23588)
Supplement: Supplementary file 1 — Table S1 The incidence of clinical outcomes in patients with or without CKD, stratified by the HRPR on clopidogrel. Table S2: Risk factors for MACEs by multivariate Cox regression analysis model. Table S3: Risk factors for HRPR on clopidogrel by univariate logistic regression analysis model Table S4: Risk factors for MACEs by univariate Cox regression analysis model. [file CLC-44-789-s001.docx]

**Supplementary table 1.** Risk factors for HRPR on clopidogrel by univariate logistic regression analysis model

| Variable | Unadjusted OR (95% CI) | p value |
| --- | --- | --- |
| Age > 65 (years) | 2.28(1.32-3.94) | 0.003 |
| Female gender | 3.03(1.87-4.90) | <0.001 |
| BMI > 30 (kg/m^2^) | 0.68(0.35-1.35) | 0.275 |
| Current smoking | 1.13(0.68-1.90) | 0.636 |
| Hypertension | 2.06(1.05-4.05) | 0.035 |
| Diabetes mellitus | 1.55(0.97-2.50) | 0.070 |
| Hyperlipidemia | 1.29(0.78-2.15) | 0.326 |
| Prior stroke | 1.58(0.95-2.64) | 0.079 |
| Prior MI | 1.47(0.83-2.61) | 0.188 |
| Prior PCI | 1.15(0.68-1.98) | 0.601 |
| Prior CABG | 0.20(0.03-1.50) | 0.117 |
| Prior bleeding | 0.74(0.32-1.71) | 0.488 |
| Hemoglobin < 100 (g/L) | 2.76(1.52-5.01) | 0.001 |
| Platelet (10^9^/L) | 1.00(0.98-1.01) | 0.603 |
| Mean platelet volume (fL) | 1.14(0.96-1.35) | 0.125 |
| LDL-C (mmol/L) | 1.04(0.82-1.32) | 0.763 |
| CKD | 3.56(2.16-5.86) | <0.001 |
| LVEF < 50 (%) | 1.06(0.63-1.80) | 0.827 |
| Presentation |  |  |
| Unstable angina | Reference |  |
| NSTEMI | 0.96(0.55-1.70) | 0.894 |
| STEMI | 1.16(0.67-2.04) | 0.594 |
| PCI | 0.76(0.47-1.25) | 0.278 |
| Medication at discharge |  |  |
| ACEI or ARB | 0.63(0.39-1.01) | 0.057 |
| β-blocker | 0.94(0.50-1.77) | 0.850 |
| Statins | 0.20(0.04-1.02) | 0.053 |
| CCB | 1.32(0.79-2.22) | 0.291 |
| PPIs | 1.50(0.90-2.51) | 0.119 |

BMI, body mass index; MI, myocardial infarction; PCI, percutaneous coronary intervention; CABG, coronary artery bypass grafting; LDL-C, low-density lipoprotein cholesterol; CKD, chronic kidney disease; LVEF, Left ventricular ejection fraction. NSTEMI, non-ST-segment elevation myocardial infarction; STEMI, ST-segment elevation myocardial infarction; ACEI, angiotensin-converting enzyme inhibitor; ARB, angiotensin receptor blocker; CCB, calcium-channel blocker; PPIs, proton pump inhibitors.

**Supplementary table 2.** The incidence of clinical outcomes in patients with or without CKD, stratified by the HRPR on clopidogrel.

| Event | Non-CKD  (n=282) | | |  | CKD  (n=212) | | |
| --- | --- | --- | --- | --- | --- | --- | --- |
|  | Non-HRPR  (n=255) | HRPR  (n=27) | p value |  | Non-HRPR  (n=154) | HRPR  (n=58) | p value |
| MACEs | 13(5.1%) | 5(18.5%) | 0.020 |  | 29(18.8%) | 22(37.9%) | 0.004 |
| All-cause death | 2(0.8%) | 1(3.7%) | 0.261 |  | 18(11.7%) | 10(17.2%) | 0.287 |
| Ischemic stroke | 3(1.2%) | 3(11.1%) | 0.013 |  | 3(1.9%) | 2(3.4%) | 0.616 |
| MI | 8(3.1%) | 1(3.7%) | 0.601 |  | 8(5.2%) | 10(17.2%) | 0.010 |
| Bleeding | 5(2.0%) | 1(3.7%) | 0.456 |  | 12(7.8%) | 2(3.4%) | 0.359 |

CKD, chronic kidney disease; HRPR, high residual platelet reactivity; MACEs, major adverse clinical events; MI, myocardial infarction.

**Supplementary table 3.** Risk factors for MACEs by univariate Cox regression analysis model.

| Variable | Unadjusted HR (95% CI) | p value |
| --- | --- | --- |
| Age (per 10 years) | 1.47(1.17-1.85) | 0.001 |
| Female gender | 1.41(0.88-2.27) | 0.153 |
| BMI (kg/m^2^) | 0.95(0.88-1.02) | 0.131 |
| Current smoking | 0.76(0.43-1.33) | 0.334 |
| Hypertension | 2.48(1.14-5.42) | 0.023 |
| Diabetes mellitus | 1.53(0.94-2.48) | 0.086 |
| Hyperlipidemia | 1.26(0.75-2.11) | 0.390 |
| Prior stroke | 1.92(1.17-3.13)) | 0.010 |
| Prior MI | 2.00(1.19-3.36) | 0.009 |
| Prior PCI | 1.24(0.74-2.09) | 0.414 |
| Prior CABG | 1.45(0.86-2.45) | 0.162 |
| Prior bleeding | 1.08(0.49-2.36) | 0.848 |
| Hemoglobin < 100 (g/L) | 1.30(0.67-2.55) | 0.438 |
| Platelet (10^9^/L) | 0.98(0.99-1.00) | 0.195 |
| Mean platelet volume (fL) | 1.01(1.00-1.01) | 0.061 |
| LDL-C (mmol/L) | 1.06(0.81-1.40) | 0.659 |
| CKD | 4.16(2.43-7.13) | <0.001 |
| HRPR | 3.28(2.02-5.31) | <0.001 |
| LVEF < 50 (%) | 1.55(0.94-2.54) | 0.084 |
| Clinical presentation |  |  |
| Unstable angina | Reference |  |
| NSTEMI | 1.67(0.98-2.84)) | 0.058 |
| STEMI | 0.82(0.43-1.57) | 0.551 |
| PCI | 0.65(0.36-1.16) | 0.146 |
| Medication at discharge |  |  |
| ACEI or ARB | 0.89(0.73-1.10) | 0.278 |
| β-blocker | 1.15(0.59-2.25) | 0.686 |
| Statins | 0.32(0.10-1.01) | 0.051 |
| CCB | 1.29(0.77-2.15) | 0.337 |
| PPIs | 1.60(0.94-2.72) | 0.081 |

BMI, body mass index; MI, myocardial infarction; PCI, percutaneous coronary intervention; CABG, coronary artery bypass grafting; LDL-C, low-density lipoprotein cholesterol; CKD, chronic kidney disease; HRPR, high residual platelet reactivity; LVEF, Left ventricular ejection fraction. NSTEMI, non-ST-segment elevation myocardial infarction; STEMI, ST-segment elevation myocardial infarction; PCI, percutaneous coronary intervention; ACEI, angiotensin-converting enzyme inhibitor; ARB, angiotensin receptor blocker; CCB, calcium-channel blocker; PPIs, proton pump inhibitors.

HR, hazard ratio; CI, confidence interval;

**Supplementary table 4.** Risk factors for MACEs by multivariate Cox regression analysis model.

| Variable | Adjusted HR (95% CI) | p value |
| --- | --- | --- |
| Age (per 10 years) | 1.29(1.01-1.65) | 0.039 |
| Hypertension | 1.46(0.66-3.25) | 0.348 |
| Prior MI | 1.77(1.05-2.98) | 0.032 |
| Prior stroke | 1.37(0.83-2.25) | 0.214 |
| PPIs | 1.01(0.57-1.76) | 0.987 |
| HRPR | 2.06(1.24-3.41) | 0.005 |
| CKD | 2.88(1.63-5.10) | <0.001 |

MACEs, major adverse clinical events; MI, myocardial infarction. HRPR high residual platelet reactivity; CKD, chronic kidney disease. PPIs, proton pump inhibitors.

HR, hazard ratio; CI, confidence interval;

Multivariate cox regression analysis model included age, hypertension, prior MI, prior stroke, PPIs, HRPR, and CKD.
